# Self-organized criticality in geophysical turbulence

**DOI:** 10.1038/s41598-019-39869-w

**Published:** 2019-03-06

**Authors:** W. D. Smyth, J. D. Nash, J. N. Moum

**Affiliations:** College of Earth, Ocean and Atmospheric Sciences Oregon State University, Corvallis, Oregon USA

## Abstract

Turbulence in geophysical flows tends to organize itself so that the mean flow remains close to a stability boundary in parameter space. That characteristic suggests self-organized criticality (SOC), a statistical property that has been identified in a range of complex phenomena including earthquakes, forest fires and solar flares. This note explores the relationship between the properties of forced, sheared, stratified turbulence (as found in oceans, atmospheres and other geophysical fluids) and those of SOC. Self-organization to the critical state is demonstrated in a wide range of cases drawn mostly (but not entirely) from *in situ* observations of ocean turbulence. Turbulent events in the ocean also exhibit a second characteristic associated with SOC: their sizes follow a power-law distribution indicating self-similarity. These results suggest SOC as a new conceptual foundation for the study of geophysical turbulence, an explanation for the mixing efficiency of ocean turbulence and a potential for cross-fertilization with other areas of geophysics.

## Introduction

Fluid turbulence has been a source of fascination for centuries, and typifies the highly complex phenomena that are common in nature but poorly described by classical physics. Geophysical fluids - the mantle, oceans, atmosphere and extraterrestrial plasmas - are invariably turbulent. Because turbulence dominates transports of flow properties, it is critical that we understand and predict its effects. Kolmogorov’s (1941) model of stationary, homogeneous, isotropic turbulence is simple enough that we can develop intuition and derive useful formulas e.g.^1^. In nature, however, turbulence is neither stationary, nor homogeneous, nor isotropic, and Kolmogorov’s results are correspondingly approximate. Despite this limitation, the theory provides a conceptual foundation from which to explore the real world, the most interesting aspects being the ways in which reality departs from Kolmogorov.

Two primary factors that underlie the differences between geophysical turbulence and Kolmogorov’s idealization are stratification and shear (i.e., vertical variations of density and horizontal velocity, respectively), both of which impose a preferred direction on the dynamics. To better understand geophysical turbulence it would be valuable to have a new point of departure, a theory that simplifies sheared, stratified turbulence the way that Kolmogorov did for isotropic turbulence.

This note explores the possibility that the new point of departure is to be found in the paradigm of self-organized criticality hereafter SOC^2^. In the canonical model of SOC, one imagines sand poured continuously at a point on a horizontal surface. Growing steeper at first, the sandpile eventually exceeds a critical slope and sporadic avalanches act to relieve the resulting instability. Called the ‘‘angle of repose”, the critical slope reflects the structure of the sand grains. Over time, the avalanches reach a metastable equilibrium with the sand source such that the slope is maintained, on average, at the angle of repose. Analogous behavior has been observed in a wide range of complex phenomena including earthquakes, forest fires, solar flares, stock market crashes and biological extinction events e.g.^3–6^. This ubiquity suggests a promise of cross-fertilization: an advance in understanding solar flares, for example, might furnish a clue to the prediction of earthquakes.

Stratified, parallel shear flows undergo a transition between stable and unstable states at a critical value of the gradient Richardson number, $Ri$, defined as

1 $$Ri=\frac{{\overline{B}}_{z}}{{\overline{U}}_{z}^{2}+{\overline{V}}_{z}^{2}},$$

where ${\bar{B}}_{z}$ represents the vertical buoyancy gradient and $\{{\bar{U}}_{z},{\bar{V}}_{z}\}$ the shear of the horizontal current. Overbars indicate an averaging operator (typically defined over time or horizontal distance). If some external force acts continuously to increase the shear, thereby decreasing $Ri$, then the eventual decrease below the critical value will be accompanied by instability and then by turbulence. Turbulence acts in turn to diffuse the shear and thus to redirect $Ri$ back toward the critical value. This critical $Ri$ is therefore analogous to the angle of repose in the sandpile example^7,8^. Its value is substantially independent of the forcing, and is usually not far from the inviscid upper bound for instability, namely 1/4^9,10^. Here we will review an accumulation of evidence suggesting that the tendency to maintain $Ri$ near 1/4 is a generic property of forced, stratified, parallel shear flows on geophysical scales, and therefore that the SOC paradigm is relevant.

There has been controversy over the most useful definition of SOC^11^, as may be expected when so many discipline boundaries are crossed. Aschwanden^4^ suggests the following: “… (SOC) is a critical state of a nonlinear energy dissipation system that is slowly and continuously driven towards a critical value of a system-wide instability threshold, producing scale-free, fractal-diffusive, and intermittent avalanches with powerlaw-like size distributions.” Here, “avalanches” is understood to mean sporadic, dissipative events that relieve instabilities and thereby direct the system back toward the critical state. To compare geophysical turbulence with SOC, we abstract from Aschwanden’s definition the following two criteria:

1. The flow maintains itself near a critical state without external tuning.
2. This self-regulation is due to intermittent events whose amplitude is distributed according to a power law.

Our main purpose here is to demonstrate these two properties in forced, geophysical stratified shear flow. We describe the mechanics of self-organization, and in the process gain insight into the efficiency of mixing by turbulence, another focus of controversy in recent years^12^. We close by discussing characteristics of geophysical turbulence whose relationship to SOC is less clear.

## Threshold Behavior in the Equatorial Oceans and Elsewhere

**Figure 1:**
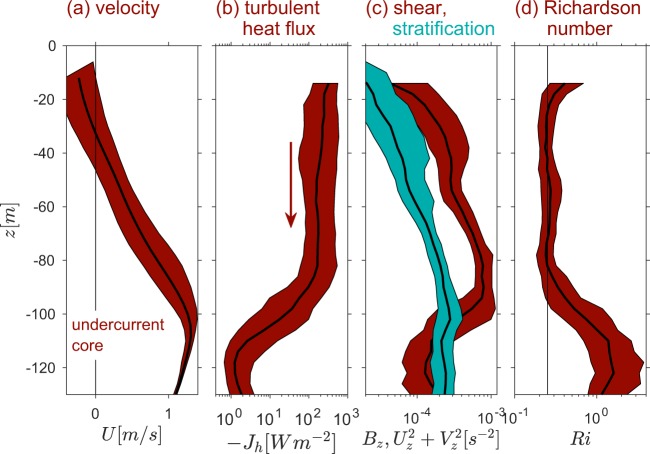
Mean flow and turbulence in the upper equatorial Pacific. Profiles show the median and the quartile range of 1-hour, 4 m bins from two weeks of shipboard measurements (cruise A in Table 1). (**a**) Eastward velocity, showing the sheared upper flank of the equatorial undercurrent. (**b**) Turbulent heat flux (positive downward). (**c**) Buoyancy gradient *B*_*z*_ (blue) and squared shear (red). (**d**) Gradient Richardson number with the vertical line at $Ri=1/4$. See^15^ for further details.

It has long been known that, on the turbulent upper flank of the Pacific equatorial undercurrent, $Ri$ remains near 1/4 most of the time^13–15^. A vertically-sheared current is maintained by the trade winds, which force westward flow at the surface and eastward return flow at, typically, 100 m depth (Fig. 1a). The resulting turbulence is an important factor in the global climate system as it carries much of the tropical solar heat flux into the ocean interior (Fig. 1b). Stable stratification is maintained against turbulent diffusion by the combination of solar heating from above and upwelling of cold water from below due to the Earth’s rotation^16^.

**Figure 2:**
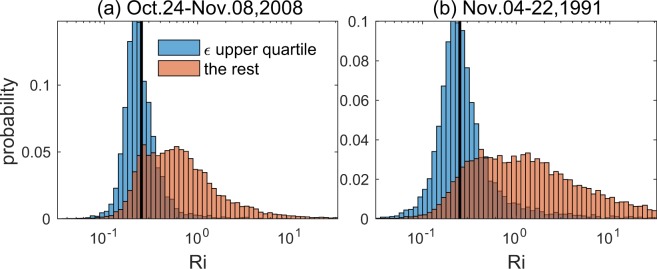
Probability density functions of $Ri$, subsampled to isolate the most turbulent time-depth regimes, showing the peak at $Ri=1/4$. Multi-week datasets were subsampled to isolate the upper quartile of turbulent heat flux values (blue). Measurements were taken on the equator at 140 W longitude. Frames (**a**) and (**b**) represent strong (La Niña) and weak (El Niño) forcing conditions, respectively.

Shear and stratification both vary considerably; their medians increase by an order of magnitude between the surface and undercurrent core (Fig. 1c). Their ratio, $Ri$ (Fig. 1d), however, is remarkably uniform over a 60 m depth range coinciding with the strongest turbulence (cf. Fig. 1b), where the time median remains conspicuously close to 1/4. Figure 2a shows the $Ri$ distribution in more detail. Depths from the base of the surface mixed layer down to 200 m are included, and the most strongly turbulent samples are distinguished by turbulent heat fluxes in the upper quartile. For those samples, the $Ri$ distribution shows a single peak around 0.2–0.25.

Figure 2a represents highly energetic La Niña conditions. In contrast, Fig. 2b shows data from the same location but a different time (boreal winter 1991–1992). An El Niño event was in progress, and the trade winds were therefore much weaker. Despite the difference in forcing, the $Ri$ distributions are similar, in particular the mode near $Ri=1/4$ that represents the strongest turbulence. Like Bak’s sandpile, therefore, the threshold state is independent of the forcing.

Other examples of turbulent stratified shear flow in which $Ri$ has been observed to remain near 1/4 include: the Atlantic equatorial undercurrent^17^, storm-driven turbulence in the North Sea^18^, a gravity current in the Mediterranean Outflow^19^, abyssal currents in the Romanche Fracture Zone^20^, stratified exchange flow in a ship canal^21^, a river plume^22^, a salt wedge estuary^23^ and clear air turbulence over Hong Kong International Airport^24^. A similar phenomenon in the stable atmospheric boundary layer is known as ‘‘global intermittency’’^25^. Examples found far from the equator indicate that the relatively parallel nature of the flow associated with the vanishing of the Coriolis effect is not critical to threshold behavior. The atmospheric example shows that fluid composition is not important. Finally, the phenomenon is not sensitive to the details of the external forcing that reduces $Ri$. Only in the Atlantic equatorial case does the forcing originate with wind stress as in the previous examples; in all other examples the forcing is different (as discussed below), but the tendency for $Ri$ to fluctuate around 1/4 is unchanged. Exceptions should be expected if the forcing is too weak to reduce $Ri$ below the critical value, or strong enough to erase the stratification entirely, or too intermittent for turbulence to have time to develop. Examples include unstable oceanic and atmospheric boundary layers.

## The Critical State and its Maintenance

How should this tendency for $Ri$ to fluctuate around 1/4 be interpreted? The observed distributions of $Ri$ suggest that flow fluctuates between regimes of turbulence growth and decay separated, roughly, by $Ri\sim 1/4$, as would be expected if turbulence is driven by shear instability. Smyth *et al*.^7,8^ have hypothesized that such fluctuations are governed by a competition between (1) external forcing (e.g., the wind), which increases shear and thereby decreases $Ri$, leading eventually to instability and turbulence, and (2) turbulent diffusion which acts to increase $Ri$, relaxing the flow back to the stable state. We now describe this interplay between forcing and diffusion in detail, together with conditions under which turbulence is expected to grow or decay.

**Figure 3:**
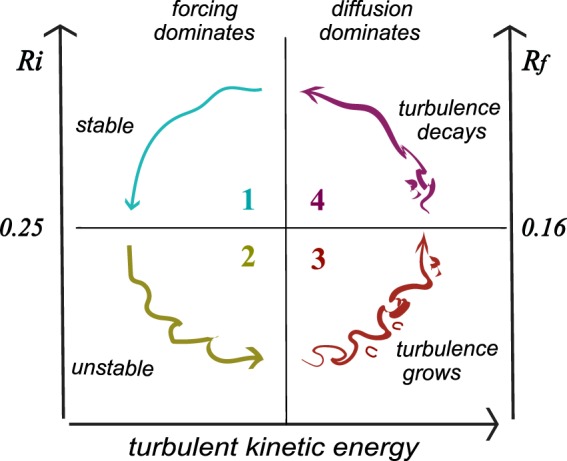
Regime diagram for fluctuations that keep stratified shear flow near the critical state. Turbulence decays (grows) in the upper (lower) half. Forcing (diffusion) dominates in the left (right) half. Time increases counterclockwise. The Richardson numbers $Ri$ and ${R}_{f}$ are related by (3,4).

The process can be understood with the aid of the regime diagram in Fig. 3. Over time, the flow state moves counterclockwise through four quadrants in which different physical factors dominate its evolution. We now describe these factors in turn.

### Forcing

At the equator, easterly trade winds force a steady westward surface current. As a result, water piles up near the western boundary, creating a pressure gradient and forcing a return current at depth, the equatorial undercurrent. The shear between these two currents appears in the denominator of $Ri$ [cf. (1)] and is accelerated by the wind; hence, the forcing acts to reduce $Ri$.

Forcing can take different forms in different regimes of geophysical turbulence. In dense gravity currents e.g.^19,23^, gravity accelerates a dense layer adjacent to the bottom while viscous stress at the bottom creates shear within the flow. In internal waves, $Ri$ is locally, often for long enough to develop turbulent events. This is a likely cause for the clear air turbulence observation^24^.

### Turbulence onset

Linear perturbation theory tells us that $Ri$ must be reduced to values below 1/4 before normal mode instability can begin transferring energy from the mean flow to the disturbance (the Miles-Howard theorem^9,10^). The proof involves exacting assumptions that are implausible in geophysical turbulence: the initial flow must be inviscid, nondiffusive, steady and parallel. In fact, geophysical instabilities invariably grow in the presence of some degree of small-scale turbulence, so that the Miles-Howard assumptions are formally invalid. When reasonable assumptions are made about pre-existing turbulence, the upper bound on $Ri$ varies by a few tens of percent^26^. From a pragmatic standpoint, though, the connection between $Ri < 1/4$ and the onset of shear-driven turbulence is well established e.g.^27^.

### Turbulent diffusion

The diffusive action of sheared turbulence in stratified flow tends to reduce gradients of buoyancy and velocity, usually at similar rates. The result is an increase of $Ri$, a mathematical consequence of the fact that the numerator of $Ri$ is a gradient while the denominator is a squared gradient [recall (1)]. As a simple example, consider a horizontal layer of turbulent fluid with uniform mean gradients ${U}_{z}$ and ${B}_{z}$. Now suppose that, over time, turbulent entrainment thickens this layer by a factor $a$, so that ${U}_{z}$ and ${B}_{z}$ are decreased by the same factor. In that case, $Ri={B}_{z}/{U}_{z}^{2}$ must also increase by the factor *a*.

### Turbulence collapse and the efficiency of mixing

Under what conditions should we expect turbulence to decay? Because sheared, stratified turbulence is sustained by the transfer of kinetic energy from the mean flow, we might expect that turbulence will decay when that transfer becomes impossible. Based on what we’ve seen so far, a reasonable guess is that this happens when $Ri$ increases to values exceeding ~1/4^9,10^.

More generally, the decay of turbulence is predicted using a variant of $Ri$ called the flux Richardson number:

2 $${R}_{f}=\frac{\overline{b^{\prime} w^{\prime} }}{{\overline{U}}_{z}\overline{u^{\prime} w^{\prime} }+{\overline{V}}_{z}\overline{v^{\prime} w^{\prime} }},$$

where primed lower-case letters denote departures from the mean state. ${R}_{f}$ may also be thought of as the mixing efficiency, i.e., the fraction of energy input from the mean flow [represented by the denominator of (2)] that goes into work against gravity (the numerator), e.g.^28,29^. An estimate of the critical value of ${R}_{f}$ follows from the intuition that, if turbulence is required to do too much work against gravity, it cannot be sustained. Ellison^30^, based on a combination of energy conservation arguments and empirical observations, estimated the maximum ${R}_{f}$ for the maintenance of turbulence to be 0.15. Observational, numerical and lab-based estimates of ${R}_{f}$ are typically 0.15–0.2^28,31–34^, though values from zero to ~0.4 are found in specific cases^12,35–37^.

The collapse criterion can be related to the onset criterion via consideration of the turbulent Prandtl number $P{r}_{t}={K}_{m}/{K}_{h}$, where where ${K}_{m}$ and ${K}_{h}$ are eddy coefficients for momentum and heat such that $\overline{w^{\prime} T^{\prime} }=-\,{K}_{h}{\bar{T}}_{z}$, $\overline{u^{\prime} w^{\prime} }=-\,{K}_{m}{\bar{U}}_{z}$ and $\overline{v^{\prime} w^{\prime} }=-\,{K}_{m}{\bar{V}}_{z}$. Substituting these definitions into (2) yields

3 $${R}_{f}=\frac{Ri}{P{r}_{t}}.$$

In unstratified turbulence, we know empirically that momentum and heat mix similarly, the so-called Reynolds analogy; hence $P{r}_{t}\sim 1$. Stratified turbulence, however, is a combination of classical, isotropic turbulence and internal gravity waves^38^, and the latter component carries no heat. As a result we should expect $P{r}_{t}$ to increase. These notions have been confirmed and made quantitative in a variety of studies; e.g., Esau and Grachev^39^ collected data from diverse sources and showed that all were fit by

4 $$P{r}_{t}=0.8+3.0Ri.$$

A similar but independent parameterization has been given by Venayagamoorthy *et al*.^40^.

At the beginning of this subsection we hypothesized that collapse occurs when $Ri$ exceeds 1/4. In (4), $Ri=1/4$ corresponds to $P{r}_{t}=1.5$, and therefore via (3) to ${R}_{f}=0.16$. (As a measure of the uncertainty, the Venayagamoorthy formula^40^ gives $P{r}_{t}=1.2$, and therefore ${R}_{f}=0.2$). These values, corresponding to the onset condition $Ri\sim 0.25$, are consistent with the theoretical/empirical estimates listed above^12,27,30–36^; hence the hypothesis that turbulence collapse begins when the transfer of energy from the mean flow stops is consistent with our existing understanding of mixing efficiency in oceanic turbulence.

#### Summary: fluctuations around the critical state

The preceding results suggest that $Ri\sim 0.25$, ${R}_{f}\sim 0.16$ is a boundary between regimes of growing and decaying turbulence in forced stratified shear flow. One can easily imagine that turbulence near this boundary is highly intermittent. Moreover, the mixing action of turbulence can cause the flow to remain near this regime over a wide range of forcing strengths. Referring to Fig. 3, this process of self-organization may be summarized as follows.

- Throughout the upper half-plane, there is no normal mode growth (because $Ri > 0.25$), and any turbulence that is present is decaying (because ${R}_{f} > 0.16$). Turbulence decay moves the flow into quadrant 1 (upper left).
- In quadrant 1, diffusion is weak enough that forcing dominates, reducing $Ri$ and ${R}_{f}$ and moving the flow to quadrant 2.
- In quadrant 2, normal mode instability creates new turbulence, while any turbulence already present can grow stronger by extracting energy from the mean flow.
- The growth of turbulence moves the flow into quadrant 3, where the mixing action of the turbulence overcomes the forcing and thus increases $Ri$ and ${R}_{f}$.
- Diffusion moves the flow to quadrant 4, where turbulence begins to decay and the cycle repeats.

This process is a close analogue of the Bak^2^ sandpile model. The forcing (e.g., the trade winds) corresponds to the sand source, and the sporadic mixing events to the avalanches. The “angle of repose” for stratified shear flow is the critical state $Ri\sim 0.25$. This process is evident over a wide range of forcing scenarios as reviewed above, i.e., no external tuning is needed to maintain the flow near the critical state. Criterion #1 for SOC is therefore satisfied.

## Power-Law Scaling of Event Sizes

The power-law size distribution (criterion #2 above) is a standard characteristic of “avalanches” in SOC systems, e.g., the Gutenberg-Richter law of earthquake frequency^41^. Power laws are also familiar in Kolmogorov’s isotropic turbulence, e.g., the inertial and inertial-convective subranges of the kinetic energy and scalar variance spectra. These do not indicate SOC, however, because no critical state is involved.

To test for this characteristic, we assembled a database of 211,202 turbulent overturns observed between the base of the surface mixed layer and 200 m depth. To maximize both the number of events and the range of equatorial surface forcing and vertical ocean structure regimes, we combined 9471 profiles from three cruises (Table 1). Centimeter-resolving density profiles were searched for layers of static instability which were identified as overturns^42,43^ (see Supplementary Material for details). The overturn height *L* is used here as a measure of the “avalanche” size.

**Table 1:** Microstructure datasets from the equatorial Pacific used in Figs 4 and 5.

Label	Longitude	Dates	Reference	Profiles	Overturns
A	140 W	25 Oct.–8 Nov. 2008	^51^	2476	23,628
B	140 W	4 Nov.–24 Nov. 1991	^52^	3907	98,211
C	110 W	15 Nov.–3 Dec. 2014	^53^	3088	89,363

**Figure 4:**
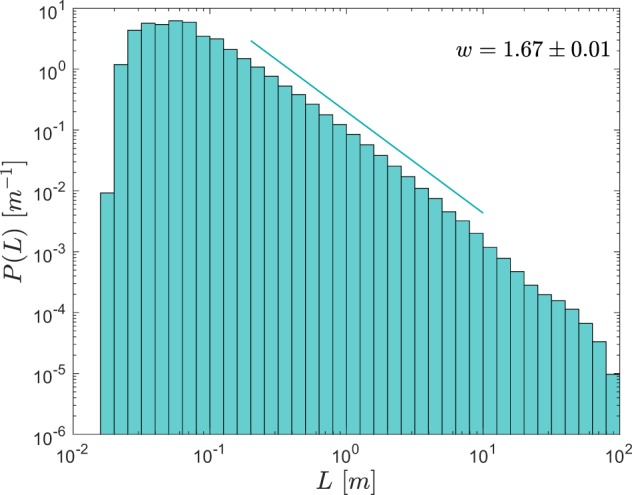
Probability density function $P$ of the vertical scale $L$ of turbulent overturns in the upper equatorial Pacific. Overturns were identified on the equator between the mixed layer base and 200 m depth using microstructure data from three equatorial cruises (Table 1). $P$ is defined such that ${\int }_{0}^{\infty }\,PdL=1$. Straight line with slope *w* shows the maximum likelihood estimate of the exponent^44^, given numerically in the legend, and the range over which it was computed.

The probability density function $P(L)$ approximates the power-law form *P*~${L}^{-w}$, where *w* is a constant, remarkably well over nearly three decades (Fig. 4). Events exceeding the thickness of the marginally unstable layer (about 60 m in the case shown in Fig. 1) are rare, imposing a large-size cutoff on the intermediate range. The rolloff at small scales is due to the finite vertical resolution of the measurements, which are spaced at about 0.5 cm and do not resolve overturns smaller than about 2 cm. Inasmuch as $P(L)$ is linear between these extremes (Fig. 4), criterion #2 for SOC is satisfied.

The power-law exponent, calculated using the maximum likelihood method^44^, is $w=1.67\pm 0.01$. While the similarity between *w* and the Obukhov-Corrsin slope for temperature variance in the inertial subrange −5/3^45,46^, is striking, we suspect it is fortuitous. (Whereas a temperature spectrum describes a superposition of disturbances on all scales, a large overturns engulfs all smaller overturns within it so that they do not show up separately in this measurement).

**Figure 5:**
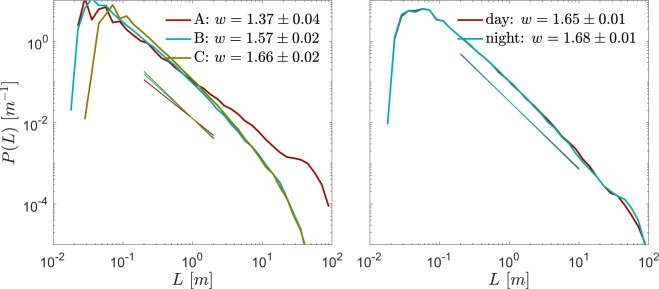
Probability density function $P$ of the vertical scale $L$ of turbulent overturns in the upper equatorial Pacific. Overturns were subsampled by (**a**) cruise (details in Table 1) and (**b**) day (local time 06:00–18:00)/night (18:00–06:00). Straight lines show the maximum likelihood estimate of the exponent^44^, given numerically in the legend.

To explore this behavior further, the dataset is subsampled in two ways (Fig. 5). First, power-law exponents for the three individual cruises are 1.37 ± 0.04 and 1.57 ± 0.02 and 1.66 ± 0.02. The power law form is observed over a smaller range of sizes in these subsamples. In contrast, dividing the diurnal cycle into daytime and nighttime hours (Fig. 5b) gives nearly identical *w*-values.

Sanchez and Newman^47^ have proposed a more specific constraint on the SOC paradigm, namely that there be no characteristic length scale (other than the smallest and largest scales at which the SOC physics operates). In all cases considered here, $0 < w < 2$, indicating that the expectation value $\langle L\rangle ={\int }_{0}^{\infty }\,L\,P(L)dL$ diverges or, more precisely, would diverge if *L* were not limited by the precision of the measurements and the size of the system (~60 m). The scale-free constraint is therefore satisfied.

Power law scaling expresses the absence of a characteristic length scale, a property of turbulence that bedevils parameterization efforts. In most geophysical turbulence, there is no controlling length scale except for the size of the domain in which the phenomenon is observed (~60 m in the case of the equatorial undercurrent).

## Differences Between Geophysical Turbulence and SOC

Many SOC systems exhibit power law behavior not only in space but in time, and therefore have no intrinsic time scale. That is not the case with geophysical turbulence, whose intrinsic time scale can be chosen as ${({U}_{z}^{2}+{V}_{z}^{2})}^{-1/2}$ or ${B}_{z}^{-\mathrm{1/2}}$. (These choices are nearly equivalent since their ratio, $R{i}^{\mathrm{1/2}}$, does not vary much).

In the present investigation we have not considered the temporal aspect of geophysical turbulence. In the equatorial case, besides the existence of an intrinsic time scale, the diurnal cycle imposes its own time scale: conditions for SOC exist mainly at night. In addition, the driving may be insufficiently “slow” in the sense that event durations are not much shorter than the wait times between events^8,47^.

The appearance of turbulence when $Ri$ drops below 1/4 is not instantaneous; the instability grows exponentially at a rate which becomes positive when $Ri < 1/4$ (approximately) and continues to increase as $Ri$ decreases further. This lack of sharpness in the boundary between turbulence growth and decay might lead to a significant difference in behavior between forced, stratified shear flows and canonical models of SOC.

## Discussion

We have seen that forced, stratified shear flows, such as are found in the oceans and atmosphere, resemble SOC by virtue of the instability that sets in at a critical value of the gradient Richardson number. (This contrasts with Kolmogorov’s isotropic turbulence, which does not fit the SOC paradigm because the flow is not attracted to a critical state). We have also seen that probability density functions of turbulent overturn sizes approximate a power law, a second defining characteristic of SOC.

Salehipour *et al*.^48^ have shown via direct simulations that events initiated by Holmboe instability exhibit characteristics of SOC *internally*, organizing the flow so that $Ri$ remains near 1/4 throughout the volume enclosed by the event. This does not happen when KH instability initiates the turbulence. The measurements described here do not distinguish between those two mechanisms, but Salehipour *et al*.’s finding suggests that the Holmboe mechanism is more important than we have recognized e.g.^49^, as it could account for the observed SOC behavior.

Examination of the “avalanche” mechanism has yielded a side benefit - an explanation for the observed mixing efficiency of ocean turbulence. From theoretical considerations, if $Ri$ fluctuates around 1/4, ${R}_{f}$ must fluctuate around a somewhat smaller value which empirical observations identify as 0.16 (to within a few tens of percent). The more familiar form of the mixing efficiency, ${\rm{\Gamma }}={R}_{f}/(1-{R}_{f})$ is then 0.2. This value is observed over a large majority of measured oceanic regimes, an observation that has eluded explanation. We now see that ${\rm{\Gamma }}=0.2$ is the expected outcome of a threshold mechanism mediated by KH-type instabilities of stratified shear flow.

The connection with SOC suggests two major avenues for future exploration. First, like Kolmogorov’s isotropic turbulence, SOC may provide a useful conceptual picture of sheared, stratified turbulence, in the sense that important insights may be gained by looking at departures from the theory. For example, the presence of a time scale may reveal a new variant of SOC behaviour. A more complete accounting of turbulent oceanic and atmospheric flows that do and do not exhibit SOC-like characteristics, and why, could be revealing.

Second is the possibility of cross-fertilization. Any advance in earthquake prediction, for example, may suggest a corresponding advance in turbulence parameterization. Schorlemmer *et al*.^50^ have shown that, in tectonic fault systems, a difference in exponents of the Gutenberg-Richter earthquake distribution^41^ indicates a difference in the stress across the fault. Similarly, the difference between the exponents shown in Fig. 5a may represent a difference in forcing regimes. In the future we may describe turbulence in a forced, stratified shear flow as a stochastic ensemble of shear-driven overturns whose size distribution is determined by the influence of the forcing on the exponent *w*.

## Supplementary information

Supplementary information

LaTeX Supplementary File

## Data Availability

All data is available by request from the authors.
