# Supplementary material for: Self-organized criticality in geophysical turbulence
Source: Sci Rep. 2019 Mar 6;9:3747. doi: 10.1038/s41598-019-39869-w (PMC6403305; doi:10.1038/s41598-019-39869-w)
Supplement: Supplementary file 1 — Supplementary information [file 41598_2019_39869_MOESM1_ESM.pdf]

Self-organized criticality in geophysical turbulence

W.D. Smyth*, J.D. Nash and J.N. Moum
College of Earth, Ocean
and Atmospheric Sciences
Oregon State University, Corvallis, Oregon.

February 20, 2019

*Materials and correspondence: W.D. Smyth
College of Earth, Ocean and Atmospheric Sciences
Oregon State University, Corvallis, Oregon.
Telephone: (541) 737-3029;
email: smythw@oregonstate.edu

Supplementary material:Methodological details

"Chameleon", a free-falling, tethered microstructure profiler[53], is dropped and recovered about every 8 minutes, day and night, typically for 2-3 weeks at a stretch. Chameleon returns a quasi-vertical sequence of measurements taken over a span of 4-5 minutes as the instrument falls, typically to 250 m depth at a speed of ~1 m/s. Measurements are made with vertical spacing Δz, which is generally 0.5-1 cm. Measurements include microscale shear, temperature and conductivity, plus auxiliary quantities needed for quality control. Temperature and conductivity are combined to estimate salinity and density using the UNESCO equation of state for seawater[54].

The velocity profile $\{U, V\}$ is recorded acoustically, typically every five seconds. To reduce uncertainty, values are averaged into one-hour bins. The vertical resolution varies with instrument settings; here we have gridded the data in standard vertical bins of thickness 4 m. The resulting profiles are differentiated to form $\{U_z, V_z\}$ and combined with density from the microstructure profile (averaged in the same way) to form Ri. Figure 1 shows the median and quartiles of the hourly-averaged values in each depth bin. The width of the quartile range is sensitive to the sizes of the time and depth bins, but the clustering of Ri around 1/4 is not[15].

The microscale shear magnitude $s = \sqrt{u_z^2 + v_z^2}$, where u and v are Cartesian velocity components, horizontal but otherwise with unknown orientation due to the rotation of the instrument as it falls. From this we infer the dissipation rate ϵ using the isotropic approximation:

$$\epsilon = \frac{15}{4}\nu s^2. \tag{1}$$

We then estimate the turbulent buoyancy flux using the standard parameterization $J_b = 0.2\epsilon$ and multiply by density and heat capacity to convert to heat flux.

To identify turbulent overturns, we sort the density profile into monotonically increasing order and compute the vertical distance δ each fluid particle moves in the process. Layers in which δ is nonzero are identified as potential overturns. Layers separated by less than $2\Delta z$ are joined, and layers thinner than $2\Delta z$ are discarded.

This method is subject to unavoidable spikes in the salinity measurement.

Such a spike can appear as a large, spurious displacement, especially if it occurs near a region of weak stratification. Following Gargett & Garner[42], we guard against this by checking that each overturn contains roughly equal numbers of upward and downward displacements. If the ratio of positive to negative δ values is outside the range 1/4-4, the overturn is rejected. As a further fail-safe, we test to ensure that the median buoyancy gradient B_z is negative.

Note that each detected overturn samples a turbulent event at an unknown lateral location and evolutionary stage. Despite this uncertainty, statistics computed over many events are taken to be representative of the distribution of vertical scales.

In an intermediate range of lengths scales, the probability distribution $P(L)$ is proportional to L^{-w}, where w is a constant. We obtain w and its 95% confidence limit using the maximum likelihood method of[43], assuming that the power law holds in the intermediate range $0.1\text{m} < L < 20\text{m}$.

Data is available by request from the authors.

References

15. Smyth, W.D. & Moum, J.N., Seasonal cycles of marginal instability and deep cycle turbulence in the eastern equatorial Pacific ocean. *Geophys. Res. Lett.* **40**, 6181–6185 (2013).

42. Gargett, A.E. & Garner, T., Determining Thorpe scales from ship-lowered CTD density profiles. *J. Atmos. Oceanic Technol.* **25**, 1657–1670 (2008).

43. Page, R., Aftershocks and microaftershocks of the great Alaska earthquake of 1964. *Bull. Seis. Soc. Am.* **58**, 1131 – 1168 (1968).

53. Moum, J.N., Gregg, M.C., Lien, R.-C. & Carr, M.-E., Comparison of turbulence kinetic energy dissipation rate estimates from two ocean microstructure profilers. *J. Atmos. Oceanic Technol.*, **12**(2), 346–366 (1995).

54. Fofonoff, P. & Millard Jr., R.C., Algorithms for computation of fundamental properties of seawater, 1983. *Unesco Tech. Pap. in Mar. Sci.* **44** (1983).
